# Nature and Nurture: Effects of Affective Temperaments on Depressive Symptoms Are Markedly Modified by Stress Exposure

**DOI:** 10.3389/fpsyt.2020.00599

**Published:** 2020-06-30

**Authors:** Xenia Gonda, Nora Eszlári, Sara Sutori, Nikoletta Aspan, Zoltan Rihmer, Gabriella Juhasz, Gyorgy Bagdy

**Affiliations:** ^1^MTA-SE Neurochemistry and Neuropsychopharmacology Research Group, Hungarian Academy of Sciences, Semmelweis University, Budapest, Hungary; ^2^NAP-2-SE New Antidepressant Target Research Group, Hungarian Brain Research Program, Semmelweis University, Budapest, Hungary; ^3^Department of Psychiatry and Psychotherapy, Faculty of Medicine, Semmelweis University, Budapest, Hungary; ^4^Department of Pharmacodynamics, Faculty of Pharmacy, Semmelweis University, Budapest, Hungary; ^5^Pazmany Peter Catholic University, Budapest, Hungary; ^6^Janos Szentagothai Doctoral School of Semmelweis University, Budapest, Hungary; ^7^SE-NAP 2 Genetic Brain Imaging Migraine Research Group, Hungarian Brain Research Program, Semmelweis University, Budapest, Hungary

**Keywords:** affective temperaments, stress, recent life event, depression, environmental influences

## Abstract

**Background:**

Lack of proper consideration of the interaction between biological and environmental factors limits our understanding of the development of depression. Our cross-sectional study investigated whether recent stress influences the effect of affective temperaments on depressive symptoms.

**Methods:**

1015 general population participants completed the Brief Symptom Inventory to capture depressive symptoms, the List of Threatening Experiences Questionnaire to assess recent stressors, and the Temperament Evaluation of Memphis Pisa, Paris, and San Diego Autoquestionnaire to evaluate affective temperaments (TEMPS-A). Linear regression models were built to investigate the effect of temperament and stress on depression, temperament on stress, and the effect of temperament on depressive symptoms in different stress exposure groups.

**Results:**

Recent life events and anxious, depressive, cyclothymic, and hyperthymic temperaments significantly predicted depressive symptoms, and cyclothymic, and hyperthymic temperaments significantly predicted recent life event exposure. While in case of mild stress all affective temperaments except irritable predicted depression, in case of moderate exposure only the effect of depressive, cyclothymic, and hyperthymic temperament, while in the high exposure group only the effect of anxious temperament was significant.

**Limitations:**

All measures were based on self-report, and subjective impact of life events was not considered. This was a cross-sectional study with a correlational nature which does not allow for causative conclusions.

**Conclusions:**

The contribution of affective temperaments to depression is much higher compared to stress, and severity of exposure to life events influences the impact of affective temperaments on depressive symptoms, pointing to divergent pathways of emotional reactivity mediating the effects of stress on depression which can be exploited for prevention and treatment.

## Introduction

Depression is a highly prevalent condition contributing to significant subjective suffering, dysfunction and is predicted to become in the following years one of the illnesses associated with the highest burden (1). However, in spite of significant and extensive research, we still lack adequate understanding and insight on the development of this disorder, including the role of etiological factors. Depression is a multigenic and multifactorial illness with both genetic and environmental contributors to its development. While in the general population depression shows moderate heritability, with genetic variation contributing to 37%–42% of its variation (2, 3), depressogenic distal and proximal life events also occur relatively frequently every 3 to 4 years but trigger the onset of depressive symptoms in only about one fifth of those exposed to them (4), implicating that a biological-genetic predisposition is likely to be a prerequisite for the manifestation of depressogenic effects of stress. Yet the nature or nurture debate in case of depression is far from being settled, and the relative contribution of genetics and environmental effects in the etiological interaction, and whether it is different in case of different genes, stressors, or types of depression, is also not properly understood. The deeper understanding of the genetic components is hindered by the relative lack of replicable findings in both candidate gene and genome wide analytic studies (GWAS approaches) (5, 6) except for some promising novel GWAS results with sacrificing thorough phenotyping for the sake of larger study samples (7). One possible reason for the failure of genetic research in depression in spite of its significant heritability is the lack of proper consideration of interaction between heritable and environmental factors influencing depression (8).

Temperaments by definition represent the biologically determined and heritable core component of personality, and show a strong temporal stability manifesting early in development and persisting through the lifespan (9). While most theories of temperament have been devised to describe the healthy personality, the model of affective temperaments has been developed based on data from affective disorder patients and their healthy first-degree relatives (10, 11). Each of the five temperaments in the model (depressive, hyperthymic, cyclothymic, anxious, and irritable) can be considered precursors and in their more marked appearance as subclinical manifestations of affective disorders. The association between affective temperaments and affective illness, their pathoplastic role, as well as heritability and genetic determination of affective temperaments has been supported in several studies (11).

Temperaments describe emotional reactivity and also determine reaction to environmental influences, which plays a significant etiological role in depression. As gene x environment studies have shown, the effects of genetic determinants of depression in several cases are not manifested without exposure to environmental events, as the majority of depressogenic genes do not directly contribute to depression, but increase vulnerability to stress, and similarly, the effects of environmental stressors of depression may not lead to the emergence of depression without the presence of vulnerability genes (12–14). Therefore it seems possible that the effects of affective temperaments, considered to be strongly and closely determined by genetic and biological factors, on depression could be understood in its complexity only by investigating the interaction effects between affective temperament and stress on depression. However, previous studies have not looked at how exposure to stressful life events influences the effect of affective temperaments on depressive symptoms.

The aim of our present cross-sectional study was to investigate the effect of affective temperaments on depressive symptom severity in participants exposed to different levels of recent negative life events occurring in the previous year in a large general adult population.

## Methods

The reported study was part of the EU funded NewMood study (New Molecules in Mood Disorders, Sixth Framework Program of the EU, LSHM-CT-2004-503474) (15) approved by the Research Ethics Committee of the Medical Research Council, Budapest, Hungary, and carried out in accordance with the Declaration of Helsinki. All participants provided written informed consent before participating in the study. Further details about the population sample can be found in our previously published reports (16, 17).

### Population

Nonrelated, European white ethnic origin participants from the general adult population aged 18 to 60 years and recruited through general practices and advertisements from Budapest, Hungary (N = 1015) completed the Hungarian version of the NewMood questionnaire pack and were included in the present study. Inclusion criteria included voluntary participation, signing of informed consent, providing genetic material (not used in the present analysis) and returning the questionnaire pack. Inclusion was independent of any positive psychiatric anamnesis. Exclusion criteria included only withdrawn consent to participate. Details of the population and the recruitment process have been published in previous publications (16, 17). The study has been conducted in accordance with the Declaration of Helsinki, and has been approved by the local ethic committees. All participants gave written informed consent prior to participation in the study.

### Measures

Current depressive symptoms were measured by the depression items plus the additional items of the Brief Symptom Inventory (BSI), a 53-item psychological self-report scale developed as a shorter alterative to the widely used SCL-90-R to evaluate the psychological symptom status of psychiatric and medical patients and non-patients, which latter has been validated in several languages including Hungarian (18, 19). BSI has shown very good reliability including test-retest validity and internal consistency as well as good convergent validity with corresponding MMPI scales (20). The instrument evaluates 9 primary symptom dimensions as well as three Global Indexes, including the Global Severity Index (GSI), the Positive Symptom Distress Index (PSDI), and the Positive Symptom Total (PST), reflecting different aspects of psychological distress, as well as 4 additional items loading on several of the measured 9 dimensions not being unique to any single symptom dimension but reflecting core vegetative and clinical indicators (21). All items reflecting distress in each of the symptom dimensions are scored 0 to 4 from “not at all” to “extremely”. In the present analysis the Depression (DEP) dimension was used which measures a broad spectrum of symptoms of depression reflecting dysphoric affect, loss of interest, withdrawal, loss of energy, hopelessness, and feelings of futility. In our present analysis we used the calculated continuous weighted dimension score of Brief Symptom Inventory's (BSI) subscale for depression (DEP) with the addition of the additional four items, in order to assess current depression state.

We used the List of Threatening Experiences (LTE) questionnaire (22) to identify recent negative life events (RLE) related to intimate relationships, financial difficulties, illnesses/injuries, and social network problems occurring in the last year. LTE is a list of 12 major life event categories of considerable long-term contextual threat and etiological importance in relation to the development or episode onset of psychiatric disorders, with good discriminating power and excellent test-retest reliability making the instrument sufficient in etiological studies of psychological dysfunction and psychiatric disorders (23). The self-report instrument lists 12 categories of common life events which are highly likely to be threatening and the participants have to record if they have experienced the given life event in a specified previous time frame. In our study participants indicated if they encountered the given life event in the previous 2 months, last year or more than a year ago, and whether the life event still affects them. In the present analysis we used data on life events occurring in the previous year. The number of life event items was calculated and used for the initial analysis. Next the scores were grouped into three exposure severity categories (low = 0–1, medium = 2, high = 3 or more).

Affective temperaments were evaluated by the Temperament Evaluation of Memphis, Pisa, Paris, and San Diego (24), an instrument with 110 self-reported items collecting information on five affective temperament subtypes including depressive, cyclothymic, hyperthymic, irritable, and anxious temperaments predisposing to affective disorders validated in several languages including Hungarian (25). The TEMPS-A and its Hungarian version has been shown to possess good validity and reliability (24), and the affective temperaments as measured by TEMPS-A have consistently been shown to be associated with several clinical and course measures of psychiatric disorders (11). Participants have to answer yes or no indicating whether each item describing criteria of feelings and behaviors as well as emotional reactivity, cognitive, psychomotor, circadian, and social characteristics associated with each of the five affective temperaments describes them. In calculating subscale scores the sum of endorsed items in each temperament dimensions is used to yield a continuous measure. Dominant affective temperaments can also be determined as defined by a score at least 2 SD above the mean of the given population. In the present study we presented items related to each of the 5 affective temperaments in a mixed order and used the continuous approach, scores of the participants on each of the five temperament dimensions were calculated by summing the number of endorsed items and dividing it by the number of all answered items in the given dimension.

### Statistical Analyses

We used IBM SPSS Statistics 21 for statistical analyses. We ran linear regression models with enter method to test the effect of recent life events (RLE) as a continuous predictor on BSI depression score as the continuous outcome variable; to test the effect of the five affective temperaments as predictors on BSI depression score as the continuous outcome variable; and to test the effect of the five affective temperaments as predictors on recent life events as the continuous outcome variable. Age and sex were predictors in all these separate regression models.

In the next step, the population was separated into three groups according to RLE score according to low, moderate, and severe RLE exposure. In each group we ran a single linear regression model with enter method including the effect of all five temperaments (anxious, depressive, irritable, cyclothymic, and hyperthymic) as well as age and sex as predictors on BSI depression score as the continuous outcome variable in all models. The three models where all the five temperaments and age and sex were predictors were corrected for multiple testing by Benjamini-Hochberg's FDR correction yielding q-values.

## Results

### Descriptive Data and Validity of the Instruments

Descriptive data and statistics of our study population are presented in **Table 1**.

**Table 1:** Description of the study sample.

RLE exposure	n		Females (%)		Age, mean	BSI Depression, mean (SE)	TEMPS Anxious, mean (SE)	TEMPS Depressive, mean (SE)	TEMPS Irritable, mean (SE)	TEMPS Cyclothymic, mean (SE)	TEMPS Hyperthymic, mean (SE)
Low		699	484 (69%)	31.91		0.468 (0.0228)	0.256 (0.0076)	0.330 (0.0057)	0.215 (0.0064)	0.215 (0.0064)	0.464 (0.0072)
Moderate		192	140 (73%)	29.813		0.668 (0.0513)	0.294 (0.0150)	0.361 (0.0105)	0.265 (0.0141)	0.333 (0.0148)	0.470 (0.0123)
Severe		116	89 (77%)		30.802	0.978 (0.0821)	0.351 (0.0214)	0.397 (0.0155)	0.307 (0.0193)	0.379 (0.0216)	0.508 (0.0174)

BSI, Brief Symptom Inventory; TEMPS, Temperament Evaluation of Memphis Pisa Paris and San Diego; RLE, recent life events; SE, standard error.

Cronbach alpha coefficients for TEMPS-A were 0.6832 for depressive, 0.8232 for cyclothymic, 0.7775 for hyperthymic, 0.7923 for irritable and 0.8680 for anxious temperament subscales. These figures were highly similar to those reported during the validation of the Hungarian version of TEMPS-A where, with the exception of the depressive temperament with a Cronbach alpha of 0.65, Cronbach alpha coefficients for the other four subscales were between 0.75 and 0.81 which reflect satisfactory internal consistency (25).

For the depression subscale of BSI (BSI-DEP) Cronbach alpha coefficient was 0.9104, whereas for the currently used measure also containing the four additional items the Cronbach alpha coefficient was 0.9278 reflecting excellent internal consistency.

### Effects of Recent Life Events on Current Depressive Symptoms

In the total sample unseparated by recent life even exposure, recent life events significantly predicted BSI depression scores (β = 0.269, *p* < 0.001, q = 0.000) (**Table 2**) explaining a significant but relatively small portion of the variance of BSI depression scores (*R*^2^ = 0.076, F_3,1003_ = 27.371, *p* < 0.001).

**Table 2:** Effect of recent life events, age and sex on BSI depression scores.

	Unstandardized Coefficients		Standardized Coefficients	t	p	FDR q	95.0% Confidence Interval for B	
	B	Sth. Error	Beta				Lower Bound	Upper Bound
(Constant)	0.247	0.077		3.190	**0.001**		0.095	0.399
Age	0.003	0.0019	0.053	1.724	0.085	0.128	0.000	0.007
Sex	0.057	0.0458	0.038	1.242	0.214	0.214	−0.033	0.147
RLE	0.155	0.0175	0.269	8.854	**0.000**	***0.000***	0.121	0.189

Bold type denotes nominally significant (p < 0.05) values prior to correction; bold italics indicate significant p values surviving correction for multiple testing (p < 0.05, FDR q < 0.05).

### Effects of Affective Temperaments on Current Depressive Symptoms

In the total sample unseparated by life event exposure, anxious (β = 0.269, *p* < 0.001, q < 0.001), depressive (β = 0.185, *p* < 0.001, q < 0.001), cyclothymic (β = 0.285, *p* < 0.001, q < 0.001) and hyperthymic (β = −0.108, *P* < 0.001, q < 0.001) affective temperaments significantly predicted BSI depression, and these effects remained significant after correction for multiple testing. The effect of irritable temperament on BSI depression scores was not significant (β = 0.041, *p* = 0.208, q = 0.243) (**Table 3**). The five affective temperaments together predicted a significant portion of the variance of BSI depression (*R*^2^ = 0.464, F_7,999_ = 123.692, *p* < 0.001).

**Table 3:** Effect of affective temperaments, age and sex on BSI depression scores.

	Unstandardized Coefficients		Standardized Coefficients	t	p	FDR q	95.0% Confidence Interval for B					
	B	Sth. Error	Beta				Lower Bound			Upper Bound		
Age	0.001	0.002	0.023	0.966	0.334	0.334	−0.002			0.005		
Sex	−0.117	0.037	−0.078	−3.163	**0.002**	***0.003***	−0.190			−0.044		
Anxious	0.884	0.118	0.269	7.495	**0.000**	***0.000***	0.653			1.116		
Depressive	0.827	0.151	0.185	5.469	**0.000**	***0.000***	0.530			1.124		
Irritable	0.154	0.122	0.041	1.260	0.208	0.243	−0.086			0.394		
Cyclothymic	0.954	0.114	0.285	8.334	**0.000**	***0.000***	0.729			1.179		
Hyperthymic	−0.393	0.098	−0.108	−4.018	**0.000**	***0.000***	−0.586			−0.201		

Bold type denotes nominally significant (p < 0.05) values prior to correction; bold italics indicate significant p values surviving correction for multiple testing (p < 0.05, FDR q < 0.05).

### Effects of Affective Temperaments on Recent Life Events

In the unseparated sample only cyclothymic (β = 0.142, *p* = 0.002, q = 0.0014) and hyperthymic (β = 0.102, *p* = 0.002, q = 0.014) temperaments significantly predicted recent life events, with effects remaining significant after correction for multiple comparisons. However, anxious (β = 0.072, *p* = 0.130, q = 0.227), depressive (β = 0.072, *p* = 0.105, q = 0.245) and irritable (β = 0.014, *p* = 0.737, q = 0.737) temperaments had no significant effect (**Table 4**). The five affective temperaments together explained a small but significant portion of the variance of recent life events (R^2^ = 0.066, F_7,999_ = 10.073, *p* < 0.001).

**Table 4:** Effect of affective temperaments, age and sex on recent life events.

	Unstandardized Coefficients		Standardized Coefficients	*t*	p	FDR q	95.0% Confidence Interval for B	
	B	Sth. Error	Beta				Lower Bound	Upper Bound
(Constant)	0.297	0.213		1.398	0.163		−0.120	0.715
Age	−0.003	0.004	−0.023	−0.712	0.477	0.668	−0.009	0.004
Sex	0.040	0.085	0.015	0.475	0.635	0.741	−0.126	0.207
Anxious	0.410	0.271	0.072	1.517	0.130	0.227	−0.121	0.941
Depressive	0.563	0.347	0.072	1.622	0.105	0.245	−0.118	1.243
Irritable	0.094	0.280	0.014	0.336	0.737	0.737	−0.456	0.644
Cyclothymic	0.823	0.263	0.142	3.134	**0.002**	***0.014***	0.308	1.338
Hyperthymic	0.645	0.225	0.102	2.873	**0.004**	***0.014***	0.205	1.086

Bold type denotes nominally significant (p < 0.05) values prior to correction; bold italics indicate significant p values surviving correction for multiple testing (p < 0.05, FDR q < 0.05).

### Effect of Affective Temperaments on Current Depressive Symptoms in Groups with Mild, Moderate, and Severe Recent Stress Exposure

We also analyzed the effects of affective temperaments on current depression separately in the three exposure groups. The five affective temperaments predicted a significant and comparably high portion of the variance of current depression in all three exposure groups with low (R^2^ = 0.449, F_7,691_ = 80.382, *p* < 0.001), moderate (R^2^ = 0.499, F_7,184_ = 26.152, *p* < 0.001) and severe (R^2^ = 0.451, F_7,108_ = 12.695, *p* < 0.001) recent life exposure. In the group with low recent stress exposure after correction for multiple testing anxious (β = 0.266, *p* < 0.001, q < 0.001), depressive (β = 0.153, *p* < 0.001, q < 0.001), cyclothymic (β = 0.275, *p* < 0.001, q < 0.001) and hyperthymic (β = −0.145, *p* < 0.001, q < 0.001) temperaments all had a significant effect on BSI depression score while irritable temperament did not (β = 0.053, p = 0.178, q = 0.208) (**Table 5**). In the group with moderate RLE exposure after correction for multiple testing, the effect of depressive (β = 0.265, *p* = 0.001, q = 0.003), cyclothymic (β = 0.283, *p* < 0.001, q < 0.001) and hyperthymic (β = −0.132, *p* = 0.033, q = 0.046) affective temperaments on BSI depression was significant, the effect of anxious temperament was nominally significant but did not survive correction for multiple testing (β = 0.171, *p* = 0.030, q = 0.053), and irritable temperament had no significant effect (β = 0.104, p = 0.121, q = 0.141) (**Table 5**). In the group with severe RLE exposure, after correction for multiple testing only the effect of anxious temperament on BSI depression was significant (β = 0.422, *p* < 0.001, q < 0.001), while depressive (β = 0.183, *p* = 0.080, q = 0.87), cyclothymic (β = 0.285, *p* = 0.019, q = 0.066) irritable (β = −0.171, *p* = 0.157, q = 0.220) and hyperthymic (β = 0.003, *p* = 0.973, q = 0.973) temperaments had no significant effect (**Table 5**).

**Table 5:** Effect of affective temperaments, age and sex on BSI depression scores in the three recent life event (RLE) exposure groups.

	Unstandardized Coefficients		Standardized Coefficients	t	p	FDR q	95% Confidence Interval for B	
	B	Sth. Error	Beta				Lower Bound	Upper Bound
Low recent stress exposure RLE = 1								
Age	0.001	0.002	0.025	0.845	0.399	0.399	−0.002	0.005
Sex	−0.074	0.040	−0.057	−1.882	0.060	0.084	−0.152	0.003
Anxious	0.800	0.132	0.266	6.077	**0.000**	***0.000***	0.542	1.059
Depressive	0.618	0.164	0.153	3.757	**0.000**	***0.000***	0.295	0.941
Irritable	0.189	0.140	0.053	1.349	0.178	0.208	−0.086	0.463
Cyclothymic	0.868	0.129	0.275	6.699	**0.000**	***0.000***	0.613	1.122
Hyperthymic	−0.470	0.104	−0.149	−4.534	**0.000**	***0.000***	−0.673	−0.266
Moderate recent stress exposure RLE = 2								
Age	0.001	0.004	0.020	0.356	0.722	0.722	−0.006	0.009
Sex	−0.236	0.090	−0.148	−2.620	**0.010**	***0.023***	−0.414	−0.058
Anxious	0.585	0.267	0.171	2.189	**0.030**	0.053	0.058	1.112
Depressive	1.300	0.377	0.265	3.451	**0.001**	***0.003***	0.557	2.042
Irritable	0.379	0.243	0.104	1.560	0.121	0.141	−0.100	0.858
Cyclothymic	0.978	0.252	0.283	3.884	**0.000**	***0.000***	0.481	1.474
Hyperthymic	−0.552	0.257	−0.132	−2.151	**0.033**	***0.046***	−1.058	−0.046
Severe recent stress exposure RLE = 3								
Age	0.002	0.006	0.029	0.397	0.692	0.807	−0.009	0.014
Sex	−0.247	0.155	−0.119	−1.596	0.113	0.198	−0.555	0.060
Anxious	1.619	0.424	0.422	3.819	**0.000**	***0.000***	0.779	2.460
Depressive	0.969	0.548	0.182	1.768	0.080	0.187	−0.118	2.056
Irritable	−0.727	0.509	−0.171	−1.427	0.157	0.220	−1.737	0.283
Cyclothymic	1.079	0.452	0.285	2.391	**0.019**	0.066	0.184	1.974
Hyperthymic	0.014	0.418	0.003	0.034	0.973	0.973	−0.814	0.842

Bold type denotes nominally significant (p < 0.05) values prior to correction; bold italics indicate significant p values surviving correction for multiple testing (p < 0.05, FDR q < 0.05).

## Discussion

**Figure 1:**
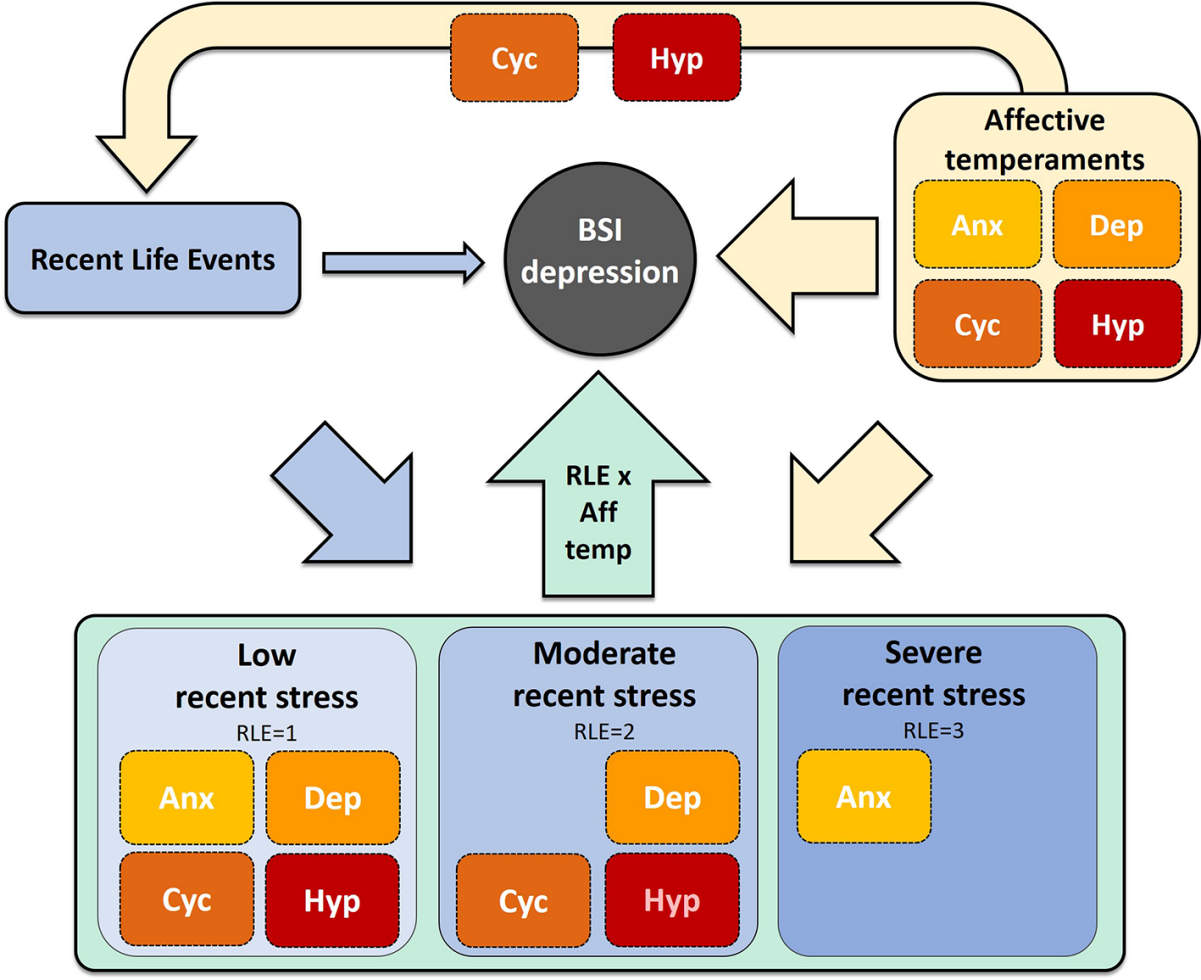
Relationship between affective temperaments, recent life event exposure and current depression severity. Aff temp, affective temperament; Anx, anxious; Cyc, cyclothymic; Dep, depressive; Hyp, hyperthymic; RLE, recent life events.

In our cross-sectional study investigating the effects of affective temperaments on current depression scores in a general adult population sample we found that affective temperaments explained an unexpectedly high portion (46.4%) of current depression scores compared to recent life events (7.8%). While in all three recent life event exposure groups the predictive value of affective temperaments was comparably high (44.9%, 49.9% and 45.1% in low, moderate, and high exposure groups, respectively), the effect of individual temperaments on current depression depended on severity of exposure to recent stress. Therefore innate emotional reactivity appears to contribute to current severity of depression with a higher magnitude than recent stress, however, the type of emotional reactivity playing the most prominent role in depression severity depends on severity to recent stress exposure (**Figure 1**).

Both independently of stress exposure, and in case of low recent life event exposure, all (depressive, cyclothymic, hyperthymic, and anxious) temperaments except for irritable temperament were significantly associated with current depression scores indicating that in the absence of recent stress, the effects of all types of emotional reactivity contribute to severity of depressive symptoms either increasing risk or protecting against it. While cyclothymic, depressive, and anxious temperaments appeared to increase risk, hyperthymic temperament was protective against current depression. However, in case of moderate recent stress exposure, only the effect of depressive and cyclothymic temperaments remained significant on current depression severity while the effects of hyperthymic temperament was marginally significant, which suggests that in case of moderately severe life events, low mood, and mood lability will be the relevant contributors to depressive symptoms. In case of severe recent stress exposure only anxious temperament was significantly associated with current depression scores. Notably, with increasing stress, the protective effect of hyperthymic temperament against depression also diminished. Age did not influence depressive symptoms in case of any level of exposure to recent life stressors, and the effect of sex was significant only in case of moderate exposure.

Besides explaining a high portion of current depression scores, affective temperaments also had a small contribution to predicting recent life events, indicating that affective temperaments determine the likelihood of encountering recent stress to a significant but small portion (6.66%). Notably, of the five affective temperaments, cyclothymic, and hyperthymic temperaments were both significantly and positively associated with recent life event exposure.

Our results add to the long debate and ever-expanding research focusing on the relative role of nature or nurture, that is, biological or environmental contributors in the development and severity of depression (26). Although genetic approaches including both candidate gene and GWAS studies generally fail to provide replicable and conclusive results (14, 27), temperaments, which develop on a strong genetic and biological basis (28), influence neuroendocrine and autonomic processes (29) and determine emotional and behavioral reactivity with an early manifestation and strong temporal stability, have been repeatedly shown to be associated with depression (30–32). While most temperamental models were developed on theoretical bases to describe the biological core of the healthy personality, specifically the model of affective temperaments was derived, besides drawing form theoretical underpinnings from Hippocrates, Kraepelin, and Kretschmer, from the observation of affective disorder patients and their healthy first-degree relatives (10, 24), The pathoplastic role of affective temperaments in affective disorders were subsequently confirmed by a large number of independent studies (11). The five affective temperaments comprising the model (depressive, cyclothymic, hyperthymic, irritable, and anxious) have also been linked to genetic variation in both healthy and patient populations (33–40) and can be considered the subclinical and subaffective manifestations of affective disorders also constituting a high-risk state for the development of these illnesses when present in a marked or dominant form (10, 41). Besides the biological contributors including temperamental traits, the risk and development of depression has also been associated with exposure to both recent and early stressors (42–46).

There appears to be a continuum in case of different manifestations of depression depending on the relative contribution of environmental and genetic determinants from more “reactive” to more “endogenous” forms, however, although with differing weight, both biological/genetic and environmental contributors are necessary for the emergence of depression which develops in the interaction of these two major factors (12–14). That is, environmental events trigger depression in those who carry a biological/genetic predisposition such as in the form of innate affective temperaments, or this biological/genetic predisposition increases sensitivity towards environmental events and leads to the manifestation of depression only in case of exposure to stress. Our present results confirm the involvement of both innate biological and outer environmental factors in current depressive severity in a general sample but also indicate that the contribution of biologically based affective temperaments is significantly larger than that of environmental influences in this specific population. Beyond both genetically determined and environmental factors being necessary for the development of depression, we have also recently reported that different genetic variation is relevant for determining severity of depression in case of differing levels of recent life event exposure (13). Our present findings that different types of affective temperaments, which likely develop based on distinct genetic backgrounds, are associated with depression given different recent stress exposure, are in line with this previous finding.

While this is the first study to investigate the effect of affective temperaments on current depression scores separately in case of different levels of stress exposure in a large general adult population sample, there have been a very few previous studies focusing on the association of stress, affective temperaments and depression in both general (47–49) and patients samples (50, 51) pointing to the mediator and moderator role of affective temperaments in the relationship between stress and depression. In a general population study, increased depressive, cyclothymic, irritable, and anxious temperaments mediated the effects of childhood traumatic life events including abuse and particularly neglect on increased depressive symptoms by enhancing the tendency to evaluate recently occurring life events as more stressful and negative (49). In another general population study depressogenic effect of recent adult negative life events was enhanced by irritable and inhibited by hyperthymic temperament supporting that affective temperaments together with both childhood abuse and adult life events have important moderator effects on depressive symptoms (48). In a similarly nonclinical sample, cyclothymic, irritable, and anxious temperaments directly worsened negative appraisal of past year life events as well as both positive and negative affects, while hyperthymic temperament showed opposite effects, suggesting in line with the other studies that early childhood traumas influence adulthood affect and emotional well-being *via* cyclothymic, irritable, and anxious temperaments (47). In major depressive disorder patients childhood neglect indirectly predicted major depressive disorder diagnosis *via* increased cyclothymic and anxious temperaments (50), while another study also in depressed patients similarly suggested that affective temperaments mediate the effect of childhood abuse on the severity of depression (51). Finally, two studies reported that affective temperaments influence severity of experienced work stress (52, 53). Thus, previous studies clearly show that increased affective temperaments, often resulting from or enhanced by early childhood traumatic experiences contribute to increased depression symptom severity both in the general population and in major depressive disorder patients, at least in part by enhancing negative appraisal of recent life events. Our study similarly found that affective temperaments have a large effect on depression scores, extending our understanding of this relationship by specifying that different affective temperaments may play a role in depressive symptom severity depending on the level of recent stress exposure.

Our study, however, differs from previous studies and is novel in several important points. First of all, our sample included a much larger sample size of more than 1000 participants. Second, previous studies focused exclusively on Asian populations, and besides previous research highlighting the geographic differences in prevalence and distribution of affective temperaments (54), it has also been suggested that depending on the cultural context affective temperaments play a differential role in contributing to depression (55). Third, previous studies either specifically focused on work stress (52, 53) or used models including both early childhood traumas and recent stress, focusing on the former (47–51) and rather than studying the effect of stress on how affective temperaments influence depression, they approached from how affective temperaments influence perception of recent stress.

Our results also lead to several important conclusions with potential clinical significance. First of all this is the first large population study in a general adult population on the role of affective temperaments in influencing severity of depressive symptoms. While we confirm that three affective temperaments, depressive, anxious, and cyclothymic increase depression also in the absence of stress, we also confirmed the protective role of hyperthymic temperament in a stress-free condition. Our results also indicate that this latter protective effect diminishes with increasing stress, and that with moderate and high stress exposure other temperaments contribute to an increase in depressive symptoms. That is, while in the relative absence of stress, intervention strategies exploiting hyperthymic temperament may be useful in decreasing depressive symptoms, these have no benefit in the face of adverse life events, and different strategies focusing on different affective temperaments may be viable prevention or intervention methods to decrease depressive symptoms among conditions characterized by different levels of stress. Thus, our results may inform the development of intervention strategies based on a combination of temperamental makeup and stress exposure.

Several limitations must be taken into consideration when interpreting the findings of our study. First of all, this was a cross-sectional study with a correlational nature which does not allow for causative conclusions. Second, our measures including temperament and depression scores and recent life events are based on self-report which may contribute to bias from several sources. Third, the categorization of life events as low, moderate or severe exposure based on the number of life events does not take into consideration the differing severity of individual life events. Also, we considered objective occurrence of life events and not their subjective meaning. Fourth, while the use of linear measurements for affective temperaments allows for the non-dichotomous attribution of participants to different temperament categories, partial multicollinearity is possible at least for some of the temperaments (e.g. depressive and hyperthymic), which may have implications in how temperaments contribute to the explanation of variance in the model. Fifth, we consider depression as a continuum of symptom severity rather than a categorical variable and thus used a general adult population sample in our study, which also included participants with lifetime major depression. Therefore further study is warranted to establish the effect of affective temperaments on depression scores in case of patients with formal diagnoses.

In conclusion, while we found that both recent stress and affective temperaments are associated with current depressive symptoms in a general adult population, we also found that the contribution of affective temperaments was much higher, and different temperaments were associated with current depression in case of different levels of stress exposure. Our findings, beyond providing insight on the role of temperaments in depression, may also pave the way for developing prevention and intervention methods based on a combination of temperamental makeup and recent stress exposure.

## Data Availability Statement

The raw data supporting the conclusions of this article will be made available by the authors, without undue reservation.

## Ethics Statement

The study protocol was reviewed and approved by Medical Research Council, Scientific and Research Ethical Review Board. The participants provided their written informed consent to participate in this study.

## Author Contributions

XG, GB, and GJ designed and conceptualized the study and collected the data. NE, SS, and NA participated in statistical analyses. All authors participated in interpreting the data. XG, NE, and SS wrote the first draft of the manuscript. SS created figures and graphical abstract. All authors participated in developing further and final versions of manuscript. All authors contributed to the article and approved the submitted version.

## Funding

This study was supported by the Sixth Framework Program of the European Union (NewMood, LSHM-CT-2004-503474); the Hungarian Academy of Sciences (MTA-SE Neuropsychopharmacology and Neurochemistry Research Group); the Hungarian Brain Research Program (grants: 2017-1.2.1-NKP-2017-00002; KTIA_13_NAPA-II/14); the National Development Agency (Grant: KTIA_NAP_13-1-2013-0001); by the Hungarian Academy of Sciences, Hungarian National Development Agency, Semmelweis University and the Hungarian Brain Research Program (Grant: KTIA_NAP_13-2-2015-0001) (MTA-SE-NAP B Genetic Brain Imaging Migraine Research Group); the ITM/NKFIH Thematic Excellence Programme, Semmelweis University; and by the SE-Neurology FIKP grant of EMMI. Xenia Gonda is supported by the Janos Bolyai Research Fellowship of the Hungarian Academy of Science. Xenia Gonda was supported by ÚNKP-19-4-SE-19 and Sara Sutori by ÚNKP-19-1-1-PPKE-63 New National Excellence Program of the Ministry for Innovation and Technology. The sponsors had no further role in the study design; in the collection, analysis and interpretation of data; in the writing of the report; and in the decision to submit the paper for publication.

## Conflict of Interest

The authors declare that the research was conducted in the absence of any commercial or financial relationships that could be construed as a potential conflict of interest.

## References

[1] Malhi GS Mann JJ Depression. Lancet (2018) 392:2299–312. 10.1016/S0140-6736(18)31948-2 30396512

[2] Flint J Kendler KS The Genetics of Major Depression. Neuron (2014) 81:484–503. 10.1016/j.neuron.2014.01.027 24507187 PMC3919201

[3] Sullivan PF Neale MC Kendler KS Genetic epidemiology of major depression: review and meta-analysis. Am J Psychiatry (2000) 157:1552–62. 10.1176/appi.ajp.157.10.1552 11007705

[4] Brown GW Bifulco A Harris TO Life events, vulnerability and onset of depression: some refinements. Br J Psychiatry (1987) 150:30–42. 10.1192/bjp.150.1.30 3651696

[5] Wray NR Pergadia ML Blackwood DHR Penninx BWJH Gordon SD Nyholt DR Genome-wide association study of major depressive disorder: new results, meta-analysis, and lessons learned. Mol Psychiatr (2012) 17:36–48. 10.1038/mp.2010.109 PMC3252611 21042317

[6] Sullivan P 96 Psychiatric Genetics Investigators. Don't give up on GWAS. Mol Psychiatr (2012) 17:2–3. 10.1038/mp.2011.94

[7] Ormel J Hartman CA Snieder H The genetics of depression: successful genome-wide association studies introduce new challenges. Transl Psychiat (2019) 9:ARTN 114. 10.1038/s41398-019-0450-5 PMC6420566 30877272

[8] Gonda X Petschner P Eszlari N Sutori S Gal Z Koncz S Effects of Different Stressors Are Modulated by Different Neurobiological Systems: The Role of GABA-A Versus CB1 Receptor Gene Variants in Anxiety and Depression. Front Cell Neurosci (2019) 13:ARTN 138. 10.3389/fncel.2019.00138 PMC6467241 31024264

[9] Bouchard TJ Genes, Environment, and Personality. Science (1994) 264:1700–1. 10.1126/science.8209250 8209250

[10] Akiskal HS Akiskal KK In search of Aristotle: Temperament, human nature, melancholia, creativity and eminence. J Affect Disord (2007) 100:1–6. 10.1016/j.jad.2007.04.013 17499855

[11] Rihmer Z Akiskal KK Rihmer A Akiskal HS Current research on affective temperaments. Curr Opin Psychiatr (2010) 23:12–8. 10.1097/YCO.0b013e32833299d4 19809321

[12] Bagdy G GJ Gonda X A new clinical evidence-based gene-environment interaction model of depression. Neuropsychopharm Hung (2012) 14:213–20. 23269207

[13] Gonda X Hullam G Antal P Eszlari N Petschner P Hokfelt TG Significance of risk polymorphisms for depression depends on stress exposure. Sci Rep (2018) 8:3946. 10.1038/s41598-018-22221-z 29500446 PMC5834495

[14] Gonda X Petschner P Eszlari N Baksa D Edes A Antal P Genetic variants in major depressive disorder: From pathophysiology to therapy. Pharmacol Therapeut (2019) 194:22–43. 10.1016/j.pharmthera.2018.09.002 30189291

[15] Deakin JF Harro J Anderson IM NewMood: a productive European model of collaboration for translational research in depression. Eur Neuropsychopharmacol (2011) 21:1–2. 10.1016/j.euroneuro.2010.11.008 21145708

[16] Juhasz G Dunham JS McKie S Thomas E Downey D Chase D The CREB1-BDNF-NTRK2 Pathway in Depression: Multiple Gene-Cognition-Environment Interactions. Biol Psychiatry (2011) 69:762–71. 10.1016/j.biopsych.2010.11.019 21215389

[17] Juhasz G Chase D Pegg E Downey D Toth ZG Stones K CNR1 Gene is Associated with High Neuroticism and Low Agreeableness and Interacts with Recent Negative Life Events to Predict Current Depressive Symptoms. Neuropsychopharmacol (2009) 34:2019–27. 10.1038/npp.2009.19 19242408

[18] Derogatis LR BSI: Brief Symptom Inventory: Administration, Scoring, and Procedures Manual National Computer Systems. Minneapolis. (1993).

[19] Unoka Z Rózsa S Kő N Kállai J Fábián Á Experiences with the Hungarian version of Derogatis Symptom Check List. Psychiatr Hung (2004) 19:235–43.

[20] Derogatis LR Melisaratos N The Brief Symptom Inventory: an introductory report. Psychol Med (1983) 13:595–605. 10.1017/S0033291700048017 6622612

[21] Derogatis LR Yevzeroff H Wittelsberger B Social class, psychological disorder, and the nature of the psychopathologic indicator. J Consult Clin Psychol (1975) 43:183–91. 10.1037/h0076514 1120828

[22] Brugha T Bebbington P Tennant C Hurry J The List of Threatening Experiences: a subset of 12 life event categories with considerable long-term contextual threat. Psychol Med (1985) 15:189–94. 10.1017/s003329170002105x 3991833

[23] Brugha TS Cragg D The List of Threatening Experiences: the reliability and validity of a brief life events questionnaire. Acta Psychiatr Scand (1990) 82:77–81. 10.1111/j.1600-0447.1990.tb01360.x 2399824

[24] Akiskal HS Akiskal KK Haykal RF Manning JS Connor PD TEMPS-A: progress towards validation of a self-rated clinical version of the Temperament Evaluation of the Memphis, Pisa, Paris, and San Diego Autoquestionnaire. J Affect Disord (2005) 85:3–16. 10.1016/j.jad.2004.12.001 15780671

[25] Rozsa S Rihmer Z Gonda X Szili I Rihmer A Ko N A study of affective temperaments in Hungary: Internal consistency and concurrent validity of the TEMPS-A against the TO and NEO-PI-R. J Affect Disord (2008) 106:45–53. 10.1016/j.jad.2007.03.016 17706791

[26] Kendler KS Ohlsson H Sundquist K Sundquist J Sources of Parent-Offspring Resemblance for Major Depression in a National Swedish Extended Adoption Study. JAMA Psychiatry (2018) 75:194–200. 10.1001/jamapsychiatry.2017.3828 29238796 PMC5838567

[27] Major Depressive Disorder Working Group of the Psychiatric GC Ripke S Wray NR Lewis CM Hamilton SP Weissman MM A mega-analysis of genome-wide association studies for major depressive disorder. Mol Psychiatry (2013) 18:497–511. 10.1038/mp.2012.21 22472876 PMC3837431

[28] Bouchard TJ McGue M Genetic and environmental influences on human psychological differences. J Neurobiol (2003) 54:4–45. 10.1002/neu.10160 12486697

[29] Buss AH The EAS theory of temperament. In: Strelau J Angleitner A, editors. Explorations in temperament : international perspectives on theory and measurement. Perspectives on individual differences. New York: Plenum Press, London; (1991). p. 43–60.

[30] Kampman O Poutanen O Can onset and recovery in depression be predicted by temperament? A systematic review and meta-analysis. J Affect Disord (2011) 135:20–7. 10.1016/j.jad.2010.12.021 21262538

[31] Elovainio M Kivimaki M Puttonen S Heponiemi T Pulkki L Keltikangas-Jarvinen L Temperament and depressive symptoms: a population-based longitudinal study on Cloninger's psychobiological temperament model. J Affect Disord (2004) 83:227–32. 10.1016/j.jad.2004.06.005 15555718

[32] Brown SL Svrakic DM Przybeck TR Cloninger CR The Relationship of Personality to Mood and Anxiety-States - a Dimensional Approach. J Psychiat Res (1992) 26:197–211. 10.1016/0022-3956(92)90023-H 1432846

[33] Rybakowski JK Dmitrzak-Weglarz M Dembinska-Krajewska D Hauser J Akiskal KK Akiskal HH Polymorphism of circadian clock genes and temperamental dimensions of the TEMPS-A in bipolar disorder. J Affect Disord (2014) 159:80–4. 10.1016/j.jad.2014.02.024 24679394

[34] Borkowska A Bielinski M Szczesny W Szwed K Tomaszewska M Kalwa A Effect of the 5-HTTLPR polymorphism on affective temperament, depression and body mass index in obesity. J Affect Disord (2015) 184:193–7. 10.1016/j.jad.2015.05.061 26093833

[35] Greenwood TA Badner JA Byerley W Keck PE McElroy SL Remick RA Heritability and genome-wide SNP linkage analysis of temperament in bipolar disorder. J Affect Disord (2013) 150:1031–40. 10.1016/j.jad.2013.05.035 PMC3759543 23759419

[36] Greenwood TA Akiskal HS Akiskal KK Study BG Kelsoe JR Genome-Wide Association Study of Temperament in Bipolar Disorder Reveals Significant Associations with Three Novel Loci. Biol Psychiat (2012) 72:303–10. 10.1016/j.biopsych.2012.01.018 PMC3925336 22365631

[37] Tsutsumi T Terao T Hatanaka K Goto S Hoaki N Wang YM Association between affective temperaments and brain-derived neurotrophic factor, Glycogen synthase kinase 3 beta and Wnt signaling pathway gene polymorphisms in healthy subjects. J Affect Disord (2011) 131:353–7. 10.1016/j.jad.2010.10.053 21115199

[38] Kawamura Y Liu XX Akiyama T Shimada T Otowa T Sakai Y The association between oxytocin receptor gene (OXTR) polymorphisms and affective temperaments, as measured by TEMPS-A. J Affect Disord (2010) 127:31–7. 10.1016/j.jad.2010.04.014 20488544

[39] Kang JI Nanikoong K Kim SJ The association of 5-HTTLPR and DRD4 VNTR polymorphisms with affective temperamental traits in healthy volunteers. J Affect Disord (2008) 109:157–63. 10.1016/j.jad.2007.12.004 18191458

[40] Gonda X Rihmer Z Zsombok T Bagdy G Akiskal KK Akiskal HS The 5HTTLPR polymorphism of the serotonin transporter gene is associated with affective temperaments as measured by TEMPS-A. J Affect Disord (2006) 91:125–31. 10.1016/j.jad.2005.12.048 16464506

[41] Akiskal HS Akiskal K Cyclothymic, hyperthymic and depressive temperaments as subaffective variants of mood disorders. In: Tasman A Riba MB, editors. Annual Review. Washington, DC: American Psychiatric Press (1992). p. 43–62.

[42] Kendler KS KarkowskiShuman L Stressful life events and genetic liability to major depression: Genetic control of exposure to the environment. Psychol Med (1997) 27:539–47. 10.1017/S0033291797004716 9153675

[43] Kendler KS Karkowski LM Prescott CA Stressful life events and major depression: Risk period, long-term contextual threat, and diagnostic specificity. J Nerv Ment Dis (1998) 186:661–9. 10.1097/00005053-199811000-00001 9824167

[44] Peterson RE Cai N Dahl AW Bigdeli TB Edwards AC Webb BT Molecular Genetic Analysis Subdivided by Adversity Exposure Suggests Etiologic Heterogeneity in Major Depression. Am J Psychiat (2018) 175:545–54. 10.1176/appi.ajp.2017.17060621 PMC5988935 29495898

[45] Kendler KS Karkowski LM Prescott CA Causal relationship between stressful life events and the onset of major depression. Am J Psychiat (1999) 156:837–41. 10.1176/ajp.156.6.837 10360120

[46] Kessler RC The effects of stressful life events on depression. Annu Rev Psychol (1997) 48:191–214. 10.1146/annurev.psych.48.1.191 9046559

[47] Kanai Y Takaesu Y Nakai Y Ichiki M Sato M Matsumoto Y The influence of childhood abuse, adult life events, and affective temperaments on the well-being of the general, nonclinical adult population. Neuropsychiatr Dis Treat (2016) 12:823–32. 10.2147/NDT.S100474 PMC4835123 27110116

[48] Nakai Y Inoue T Chen C Toda H Toyomaki A Nakato Y The moderator effects of affective temperaments, childhood abuse and adult stressful life events on depressive symptoms in the nonclinical general adult population. J Affect Disord (2015) 187:203–10. 10.1016/j.jad.2015.08.011 26342173

[49] Nakai Y Inoue T Toda H Toyomaki A Nakato Y Nakagawa S The influence of childhood abuse, adult stressful life events and temperaments on depressive symptoms in the nonclinical general adult population. J Affect Disord (2014) 158:101–7. 10.1016/j.jad.2014.02.004 24655773

[50] Toda H Inoue T Tsunoda T Nakai Y Tanichi M Tanaka T The structural equation analysis of childhood abuse, adult stressful life events, and temperaments in major depressive disorders and their influence on refractoriness. Neuropsychiatr Dis Treat (2015) 11:2079–90. 10.2147/NDT.S82236 PMC4540121 26316754

[51] Toda H Inoue T Tsunoda T Nakai Y Tanichi M Tanaka T Affective temperaments play an important role in the relationship between childhood abuse and depressive symptoms in major depressive disorder. Psychiatry Res (2016) 236:142–7. 10.1016/j.psychres.2015.12.016 26708440

[52] Tei-Tominaga M Akiyama T Miyake Y Sakai Y The relationship between temperament, job stress and overcommitment: a cross-sectional study using the TEMPS-A and a scale of ERI. Ind Health (2009) 47:509–17. 10.2486/indhealth.47.509 19834260

[53] Sakai Y Akiyama T Miyake Y Kawamura Y Tsuda H Kurabayashi L Temperament and job stress in Japanese company employees. J Affect Disord (2005) 85:101–12. 10.1016/j.jad.2004.03.012 15780681

[54] Vazquez GH Tondo L Mazzarini L Gonda X Affective temperaments in general population: a review and combined analysis from national studies. J Affect Disord (2012) 139:18–22. 10.1016/j.jad.2011.06.032 21774989

[55] Gonda X Vazquez GH Akiskal KK Akiskal HS From putative genes to temperament and culture: cultural characteristics of the distribution of dominant affective temperaments in national studies. J Affect Disord (2011) 131:45–51. 10.1016/j.jad.2010.12.003 21195481

